#  Phospholipase A2 enzymes represent a shared pathogenic pathway in psoriasis and pityriasis rubra pilaris

**DOI:** 10.1172/jci.insight.151911

**Published:** 2021-10-22

**Authors:** Shuai Shao, Jiaoling Chen, William R. Swindell, Lam C. Tsoi, Xianying Xing, Feiyang Ma, Ranjitha Uppala, Mrinal K. Sarkar, Olesya Plazyo, Allison C. Billi, Rachael Wasikowski, Kathleen M. Smith, Prisca Honore, Victoria E. Scott, Emanual Maverakis, J. Michelle Kahlenberg, Gang Wang, Nicole L. Ward, Paul W. Harms, Johann E. Gudjonsson

**Affiliations:** 1 Department of Dermatology, Xijing Hospital, Fourth Military Medical University, Xi’an, Shannxi, China.; 2 Department of Dermatology, University of Michigan, Ann Arbor, Michigan, USA.; 3 Department of Internal Medicine, the Jewish Hospital, Cincinnati, Ohio, USA.; 4 Department of Dermatology, Department of Medicine, UCLA, Los Angeles, California, USA.; 5 Abbvie Cambridge Research Center, Cambridge, Massachusetts, USA.; 6 AbbVie Dermatology Discovery, North Chicago, Illinois, USA.; 7 Department of Dermatology, UC Davis School of Medicine, Sacramento, California, USA.; 8 Division of Rheumatology, Department of Internal Medicine, University of Michigan, Ann Arbor, Michigan, USA.; 9 Departments of Nutrition and Dermatology, Case Western Reserve University, Cleveland, Ohio, USA.; 10 Department of Pathology, University of Michigan Medical School, Ann Arbor, Michigan, USA.

**Keywords:** Dermatology, Immunology, Autoimmune diseases, Innate immunity, Skin

## Abstract

Altered epidermal differentiation along with increased keratinocyte proliferation is a characteristic feature of psoriasis and pityriasis rubra pilaris (PRP). However, despite this large degree of overlapping clinical and histologic features, the molecular signatures these skin disorders share are unknown. Using global transcriptomic profiling, we demonstrate that plaque psoriasis and PRP skin lesions have high overlap, with all differentially expressed genes in PRP relative to normal skin having complete overlap with those in psoriasis. The major common pathway shared between psoriasis and PRP involves the phospholipases PLA2G2F, PLA2G4D, and PLA2G4E, which were found to be primarily expressed in the epidermis. Gene silencing each of the 3 PLA2s led to reduction in immune responses and epidermal thickness both in vitro and in vivo in a mouse model of psoriasis, establishing their proinflammatory roles. Lipidomic analyses demonstrated that PLA2s affect mobilization of a phospholipid-eicosanoid pool, which is altered in psoriatic lesions and functions to promote immune responses in keratinocytes. Taken together, our results highlight the important role of PLA2s as regulators of epidermal barrier homeostasis and inflammation, identify PLA2s as a shared pathogenic mechanism between PRP and psoriasis, and as potential therapeutic targets for both diseases.

## Introduction

Psoriasis and pityriasis rubra pilaris (PRP) have marked clinical and histological overlap, with both characterized by hyperplastic and hyperproliferative epidermis and varying degrees of dermal inflammatory infiltration (1–3). These two conditions share many molecular features such as skin barrier abnormalities (1, 4, 5), which has further led to the description of an intermediate psoriasis/familial PRP phenotype referred to as caspase recruitment domain family member 14–associated (CARD14-associated) papulosquamous eruption (4), and possibly a shared interleukin 17 (IL-17) response (6, 7). Nonetheless, some PRP cases are unresponsive to typical psoriasis treatments (8), and the overlap and potential blending between the two diseases continues to pose a diagnostic challenge. Therefore, there is a need to understand aspects of each disease at the molecular level.

Lipid metabolism has been shown to play a crucial role in epidermal barrier structural integrity and homeostasis, and regulates a variety of biological processes such as cell proliferation, angiogenesis, and immune responses (9, 10). Phospholipase A2 (PLA2) enzymes play a critical role in the release of lipid mediators via catalyzing the release of glycerophospholipids, and are involved in many physiologic processes such as eicosanoid production, host defense, and barrier function (11, 12). PLA2 enzymes are classified into 12 families that are expressed in different epidermal compartments, with each having a different substrate specificity (13), suggesting different roles for each of the 12 PLA2 enzyme families in skin homeostasis (14). The roles of PLA2 enzymes in inflammatory skin diseases are not fully understood but have been partially elucidated by recent work. Previous studies have demonstrated increased activity of cytosolic PLA2s (cPLA2s) and secreted PLA2s (sPLA2s) in psoriasis patients (15–17). Moreover, overexpression of PLA2G2F (one of the sPLA2s) in a transgenic mouse model led to development of chronic epidermal hyperplasia and hyperkeratosis, similar to the pathology of psoriasis (18). PLA2G4D, which is a cPLA2, is elevated in psoriatic mast cells and facilitates CD1a expression, which can be recognized by lipid-specific CD1a-reactive T cells, leading to production of IL-22 and IL-17A (10). Therefore, lipid metabolism and PLA2 enzymes are likely to play a pathogenic role in psoriasis.

Despite these signaling effects, the mechanism(s) by which PLA2s contribute to keratinocyte proliferation and inflammatory responses remains unknown. Here, we show that PLA2G2F (sPLA2) and PLA2G4D/PLA2G4E (cPLA2s) are highly expressed in the epidermis of both PRP and psoriasis. We further demonstrate that PLA2s contribute to the overlap between the two diseases, suggesting a shared pathogenic mechanism in terms of epithelial responses and alterations. Our findings demonstrate that PLA2s affect keratinocyte differentiation and immune responses, both in vitro and in vivo using the imiquimod-induced (IMQ-induced) psoriasis-like mouse model. In sum, our study elucidates the role of PLA2s in epidermal responses and demonstrates PLA2 inhibitors as potential therapeutic targets for hyperproliferative skin diseases.

## Results

### Genomic profiling of PRP and psoriasis identifies shared pathways involving lipid metabolism.

**Figure 1:**
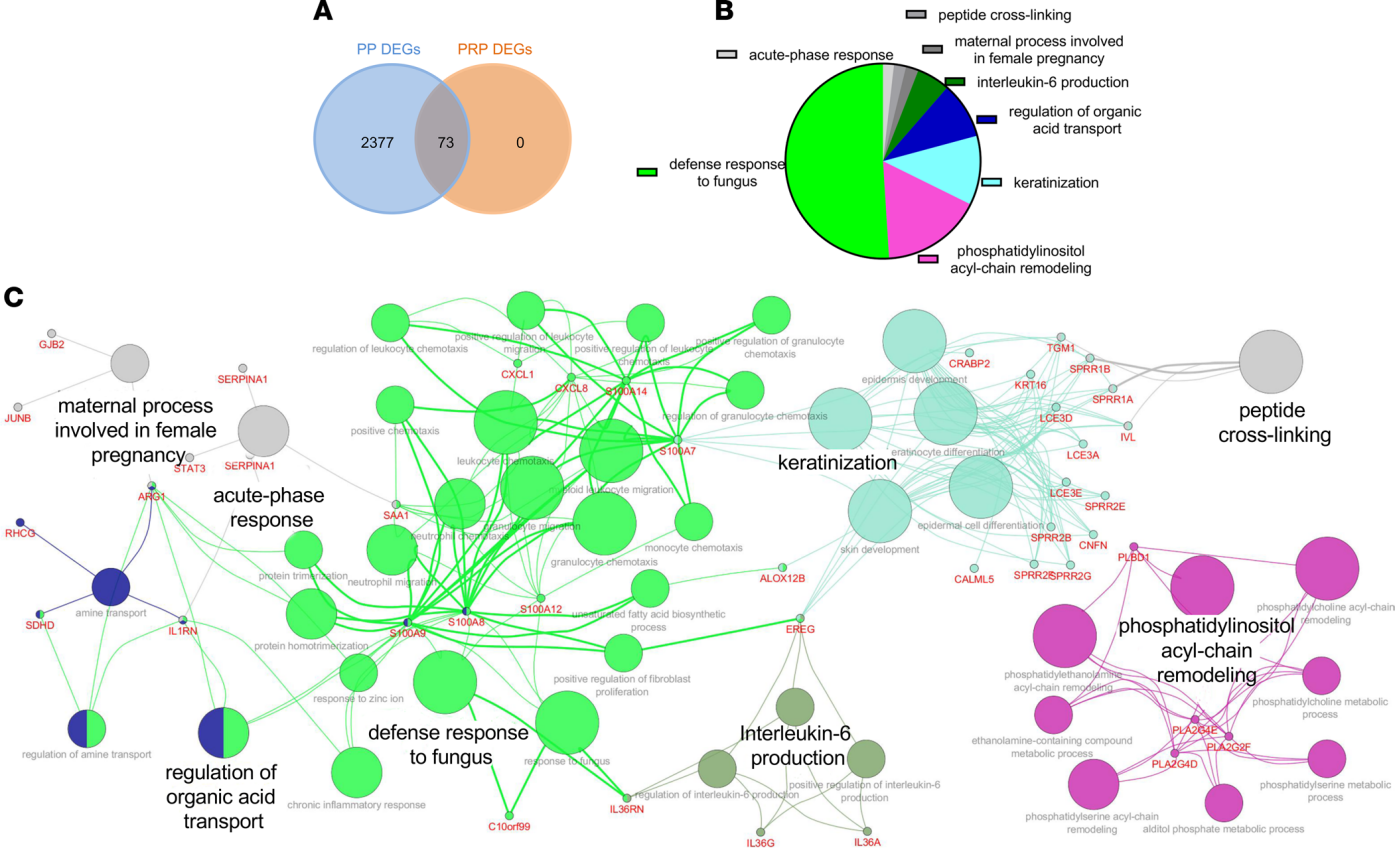
DEGs are overlapped between PRP and psoriatic lesions. (**A**) Number of genes significantly regulated in skin lesions of psoriasis (PP) and PRP patients demonstrating complete overlap of PRP genes with those of psoriasis. (**B**) Enriched Biologic Process GO terms for genes regulated consistently in psoriasis and PRP. (**C**) The metabonomics biomarker-enzyme network shared between PRP and psoriasis. DEGs, differentially expressed genes.

Given the clinical and histologic overlap between PRP and psoriasis we performed microarray profiling on RNA isolated from paraffin-embedded tissues from PRP (*n* = 13), psoriasis vulgaris patients (PP) (*n* = 22), and normal controls (NN) (*n* = 3). Principal component analysis (PCA) based on all probes and all samples revealed complete separation of psoriasis and PRP samples from control samples, with overlap between psoriasis and PRP samples (data not shown). Based on the criteria for differentially expressed genes (DEGs) of fold change (FC) of 1.5 or greater or FC of 0.5 or lower, and false discovery rate (FDR) of 0.05 or lower, we identified 2,450 DEGs in PP and only 73 DEGs in PRP skin. Importantly, all of the 73 PRP DEGs overlapped with psoriasis DEGs (Figure 1A), suggesting that PRP and psoriasis share common molecular mechanisms as drivers of the epidermal phenotype. Enriched pathways shared between psoriasis and PRP included defense response, phosphatidylinositol acyl-chain remodeling, keratinization, and IL-6 production (Figure 1B). Network analysis further showed the shared DEGs catalogued in these above pathways (Figure 1C). Furthermore, of the 73 PRP genes that overlapped between psoriasis and PRP, 36 genes were also found to be upregulated in atopic dermatitis (AD); however, the magnitude of the increase was consistently lower in AD compared with PRP or psoriasis (Supplemental Table 1; supplemental material available online with this article; https://doi.org/10.1172/jci.insight.151911DS1).

### PLA2s are overexpressed in PRP and psoriatic skin and induced by proinflammatory cytokines in keratinocytes.

**Figure 2:**
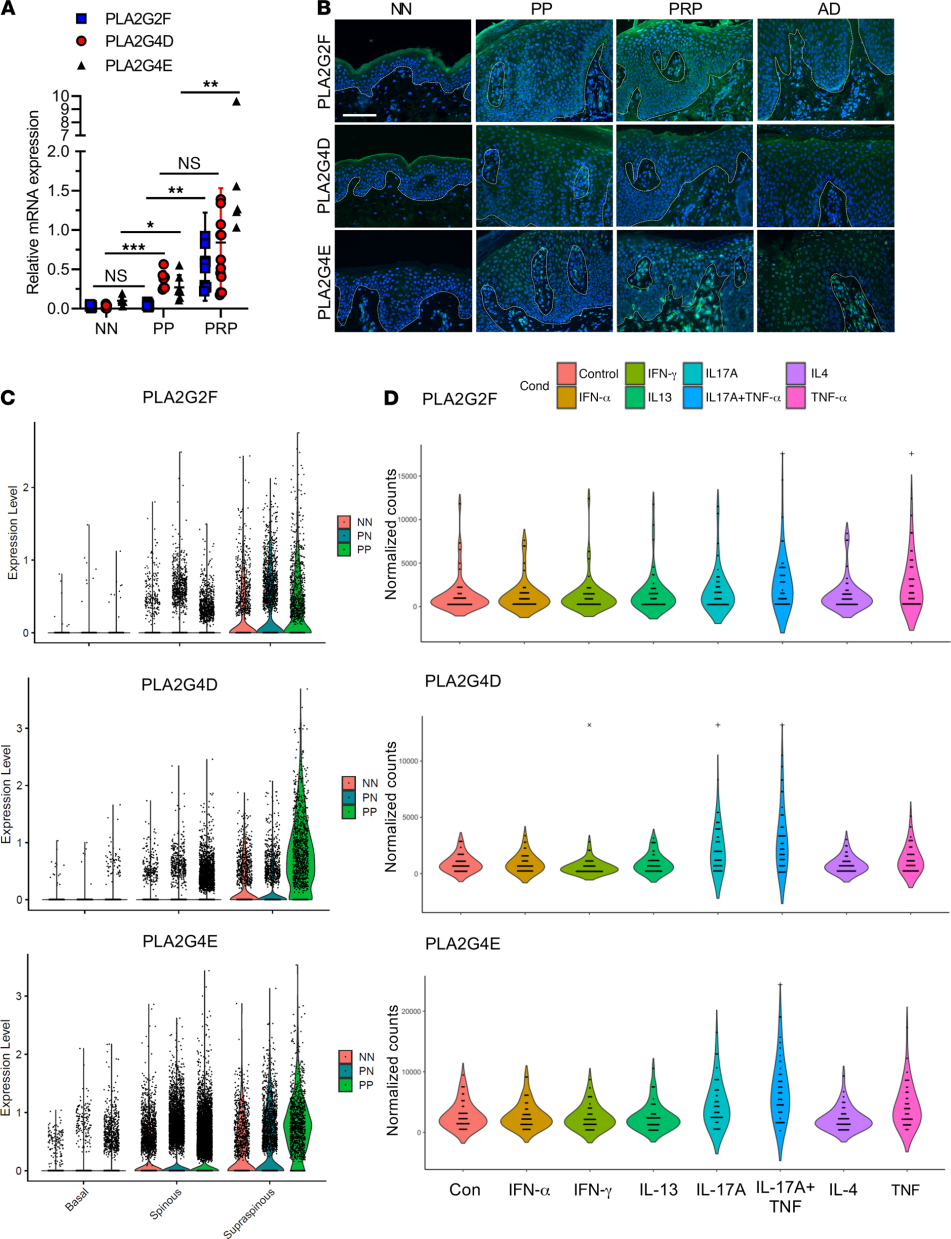
PLA2s are highly expressed in the epidermis of psoriatic lesions and TNF- and IL-17A–induced keratinocytes. (**A**) Quantitative RT-PCR of *PLA2G2F*, *PLA2G4D*, and *PLA2G4E* expression in psoriasis (PP, *n* = 8), PRP (*n* = 16), and normal controls (*n* = 8) (shown with SD). Two-way ANOVA. *P < 0.05, **P < 0.01, ***P < 0.001. NS, not significant. (**B**) Representative immunofluorescence staining of *PLA2G2F*, *PLA2G4D*, and *PLA2G4E* in skin tissues from PRP, psoriasis (PP), atopic dermatitis (AD), and normal controls (*n* = 3). Scale bar: 100 μm. (**C**) scRNA-seq analysis of psoriasis skin lesions demonstrates expression of the 3 PLA2s primarily within the 3 epidermal keratinocytes compartments. (**D**) Violin plots show the expression levels of *PLA2G2F*, *PLA2G4D*, and *PLA2G4E* in cytokine-treated keratinocytes, which were detected by RNA-seq (*n* = 38).

Among the overlapping PRP and psoriasis DEGs were several psoriasis hallmark genes, including proinflammatory cytokines and chemokines, skin barrier–related genes, and several PLA2 family genes (*PLA2G2F*, *PLA2G4D*, and *PLA2G4E*) (Supplemental Table 1). Notably, none of the PLA2 genes were found to overlap with those in AD skin (Supplemental Table 1). Quantitative reverse transcriptase polymerase chain reaction (qRT-PCR) confirmed increased gene expression of *PLA2G2F*, *PLA2G4D*, and *PLA2G4E* in PP (*n* = 8) and PRP skin (*n* = 16) compared with normal controls (*n* = 8) (Figure 2A). We further confirmed expression of *PLA2G2F* and *PLA2G4D* in other hyperproliferative and inflammatory skin disorders, including squamous cell carcinoma (Supplemental Figure 1), suggesting that expression of these PLA2s is a common pathway in diseases characterized by epithelial proliferation. Tissue immunofluorescence validated these findings, showing increased protein expression of PLA2G2F, PLA2G4D, and PLA2G4E in PP and PRP skin, but weak expression in AD skin lesions (Figure 2B). This also showed different spatial distribution in the epidermis, with PLA2G2F overexpressed throughout the epidermis, PLA2G4D primarily expressed in the keratinized/upper spinous layer, and PLA2G4E in the spinous layer and dermis (Figure 2B). To further localize PLA2 expression we assessed expression of the 3 PLA2s using single-cell RNA sequencing (scRNA-seq) data, demonstrating that these 3 PLA2s were predominantly expressed in keratinocytes, but infrequently detected in melanocytes, endothelial cells, fibroblasts, or immune cells (Supplemental Figure 2, A–C). In keratinocytes, the expression of the PLA2s was primarily found in the spinous (differentiated) and supraspinous (keratinized) epidermal layers (Figure 2C). In parallel, using data from epidermal raft cultures (Supplemental Figure 3A) we evaluated expression of the 3 PLA2 genes, which showed that expression of all 3 correlated with epidermal differentiation (Supplemental Figure 3B). In vitro, both the RNA-seq analysis (Figure 2D) and qRT-PCR results (Supplemental Figure 4) confirmed that short-term stimulation of keratinocytes with various proinflammatory cytokines increased *PLA2G2F*, *PLA2G4D*, and *PLA2G4E* mRNA expression, with tumor necrosis factor (TNF) combined with IL-17A giving the strongest induction.

### PLA2 overexpression promotes abnormal keratinocyte differentiation and inflammatory responses.

**Figure 3:**
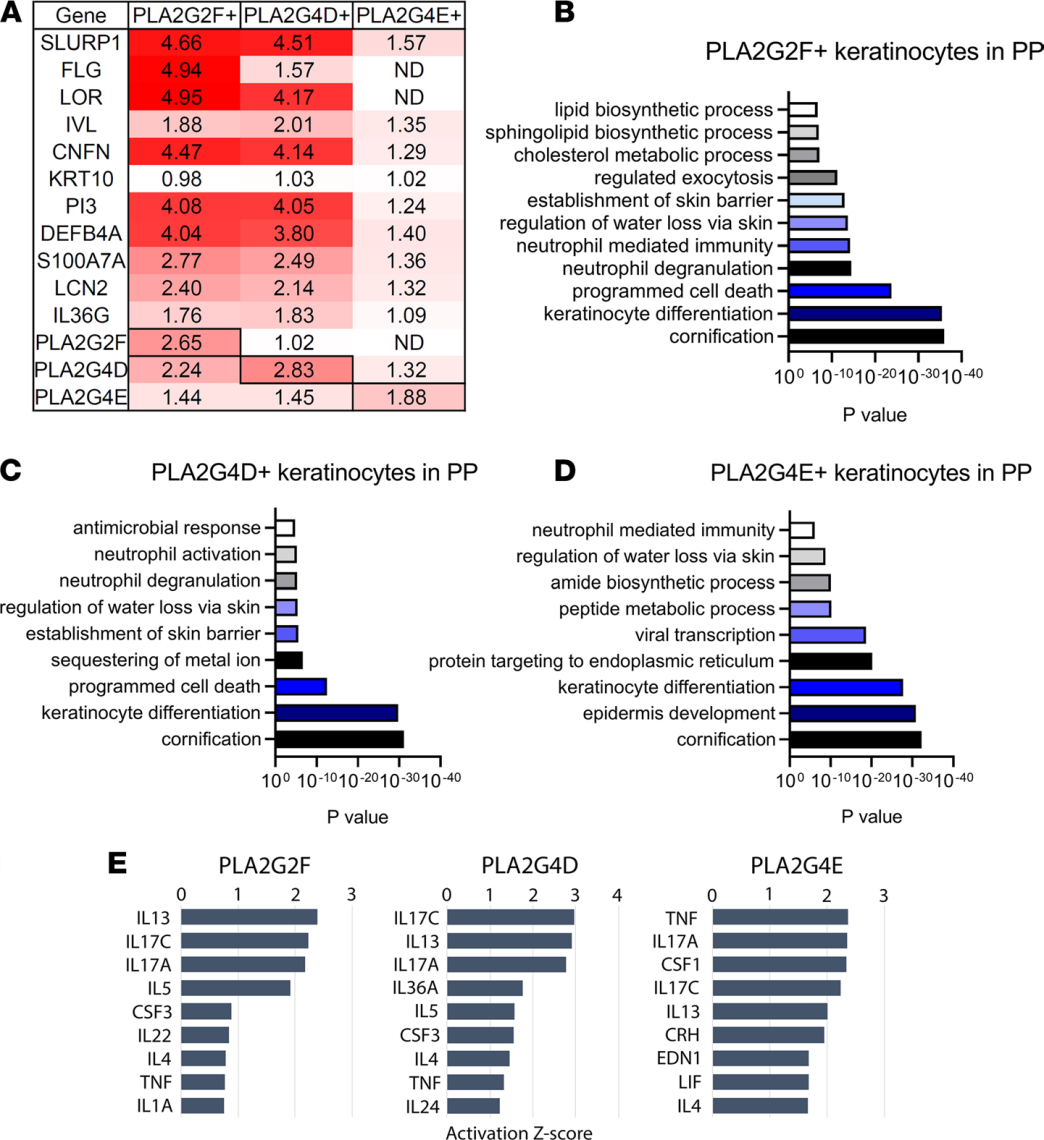
scRNA-seq analysis of *PLA2G2F*^+^, *PLA2G4D*^+^, and *PLA2G4E*^+^ keratinocytes in psoriatic lesions. (**A**) Heatmap showing genes that are expressed in *PLA2G2F*^+^, *PLA2G4D*^+^, and *PLA2G4E*^+^ keratinocytes (compared with *PLA2G2F*^–^, *PLA2G4D*^–^, and *PLA2G4E*^–^ keratinocytes, respectively) in psoriatic lesions, analyzed by scRNA-seq (*n* = 14). Changes are shown as log_2_(fold change). (**B**–**D**) GO analysis of biological process of the genes that are overexpressed in *PLA2G2F*^+^, *PLA2G4D*^+^, and *PLA2G4E*^+^ keratinocytes in psoriatic lesions. (**E**) The predicted upstream regulators for each the PLA2-positive populations. ND, not detected.

To further analyze the role of PLA2s in epidermal homeostasis, we used scRNA-seq data from psoriatic skin (*n* = 14), and compared genes that correlated with *PLA2G2F*, *PLA2G4D*, and *PLA2G4E* expression in keratinocytes in psoriatic skin against keratinocytes in the same epidermal layer that did not express any of the PLA2s. Genes identified as significantly upregulated in *PLA2G2F*^+^, *PLA2G4D*^+^, and *PLA2G4E*^+^ versus *PLA2*^–^ keratinocytes were enriched for roles in lipid metabolism, skin barrier, and inflammatory responses (Figure 3A and Supplemental Table 2). Genes upregulated in *PLA2*^+^ keratinocytes across all 3 comparisons were involved in processes such as late differentiation (*SLURP1*, *IVL*, and *CNFN*) (10, 19), lipid metabolism (*PLA2G2F* and *PLA2G4D*), and skin barrier (*DEFB4*, *S100A7A*, *LCN2*, and *IL36G*) (Figure 3A). And this regulated effect was relatively mild when comparing *PLA2G4E*^+^ versus *PLA2G4E*^–^ keratinocytes (Figure 3A and Supplemental Table 2). Enriched Gene Ontology (GO) categories in *PLA2*^+^ keratinocytes included cornification, keratinocyte differentiation, skin barrier, and also inflammatory responses such as neutrophil-mediated immunity (Figure 3, B–D). To identify upstream regulators of the gene expression changes in *PLA2*^+^ keratinocytes we used the Ingenuity Pathway Analysis tool. Among the most positive enriched upstream regulators were cytokines, including IL-17C, IL-17A, and TNF, consistent with our previous finding (Supplemental Figure 4).

**Figure 4:**
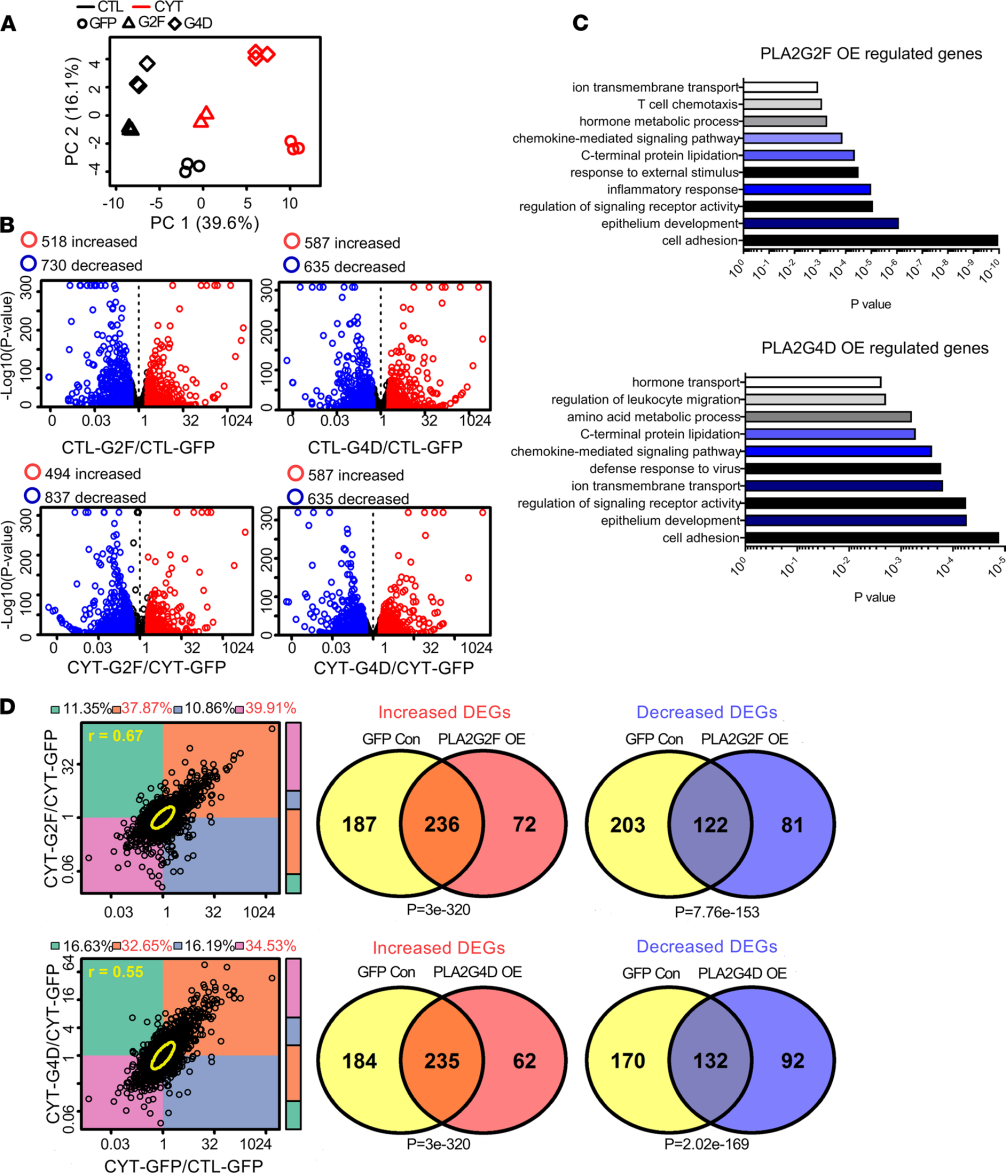
PLA2 overexpression promotes altered immune responses and keratinocyte differentiation. (**A**) Hierarchical cluster analysis of samples (CTL, control; CYT, TNF + IL-17A stimulation; G2F, *PLA2G2F* overexpression; G4D, *PLA2G4D* overexpression). (**B**) Volcano plots. The number of DEGs identified is indicated in the upper margin (red: increased DEGs; blue: decreased DEGs; FDR < 0.10; FC > 2.00 or FC < 0.50). (**C**) GO analysis of biological process of *PLA2G2F*- and *PLA2G4D*-overexpressing keratinocytes compared with controls. (**D**) Comparison of expression of genes in response to IL-17A plus TNF stimulation in WT KCs and KCs overexpressing *PLA2G2F* (upper) or *PLA2G4D* (lower). OE, overexpression; DEGs, differentially expressed genes.

To determine the direct effects of increased PLA2s, we generated keratinocytes expressing the sPLA2 enzyme PLA2G2F or the cPLA2 enzyme PLA2G4D (Supplemental Figure 5A). PCA showed clear separation between all 6 groups (*PLA2G2F* overexpressing [OE], *PLA2G4D* OE, GFP control, with and without TNF + IL-17A) (Figure 4A). Using a threshold of FDR less than 0.10 and FC greater than 2 or FC less than 0.5, we found 518 genes to be upregulated and 703 genes downregulated in *PLA2G2F*-OE keratinocytes, while in *PLA2G4D*-OE keratinocytes, 587 genes were increased and 636 genes were decreased (Figure 4B). Enriched biological processes in both included cornification, epidermal differentiation, antimicrobial humoral immune response, and inflammatory response (Figure 4C), aligning perfectly with the data from scRNA-seq from psoriatic epidermis (Figure 3). To address whether activation of PLA2s reproduces the inflammatory response induced by TNF plus IL-17A stimulation, we assessed the similarity between TNF plus IL-17A responses against *PLA2G2F*-OE and *PLA2G4D*-OE keratinocytes using RNA-seq and found a high degree of overlap (*r* = 0.67 and *r* = 0.55, respectively) (Figure 4D) both for increased and decreased DEGs (*P* = 3 × 10^–320^ and *P* = 3 × 10^–320^ for *PLA2G2F*, and *P* = 7.76 × 10^–153^ and *P* = 2.02 × 10^–169^ for *PLA2G4D*, respectively) (Figure 4D). These findings were validated by qRT-PCR for selected targets including C-C motif chemokine 20 (*CCL20*), loricrin (*LOR*), and involucrin (*IVL*) (Supplemental Figure 5, B and C).

**Figure 5:**
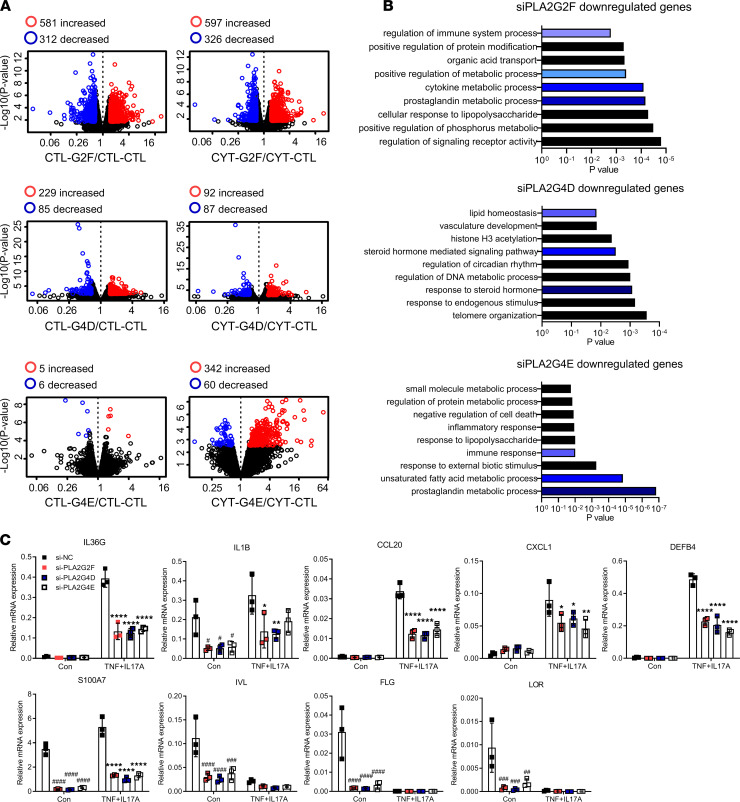
Silencing PLA2 dampens inflammatory responses and normalizes differentiation responses. (**A**) Volcano plots for each differential expression analysis in the indicated groups (CTL-G2F, *PLA2G2F*-silenced keratinocytes without any stimulus; CTL-CTL, control siRNA–transfected keratinocytes without any stimulus; CYT-CTL, control siRNA–transfected keratinocytes with TNF + IL-17A stimulation; CYT-G2F, *PLA2G2F*-silenced keratinocytes with TNF + IL-17A stimulation). (**B**) GO term analysis of biological process of the *PLA2G2F*-, *PLA2G4D*-, or *PLA2G4E*-silenced keratinocytes compared with controls. (**C**) qRT-PCR of select inflammatory genes and differentiation markers in PLA2-silenced keratinocytes with/without TNF plus IL-17A stimulation. Two-way ANOVA. Data are presented as mean ± SEM (*n* = 3). ^#^*P* < 0.05, ^##^*P* < 0.01, ^##^*P* < 0.001, ^####^*P* < 0.0001 for comparisons between control groups; *P < 0.05, **P < 0.01, ****P < 0.0001 for TNF + IL-17A groups compared with si-NC.

To address whether targeting PLA2s suppresses inflammatory responses in keratinocytes, we performed efficient siRNA-mediated knockdown for each of the 3 PLA2s (Supplemental Figure 6) with or without TNF plus IL-17A stimulation, followed by RNA-seq. DEGs were identified using a threshold of FDR less than 0.1 and a lightly lower FC thresholds than before given the lower magnitude of gene expression changes than those seen with OE (FC > 1.5 or FC < 0.67). Notably, *PLA2G2F* silencing had greater effects on gene expression than *PLA2G4D* or *PLA2G4E* silencing in unstimulated or TNF- plus IL-17A–stimulated keratinocytes (Figure 5A). Enriched biological processes across all 3 PLA2 siRNA experiments included metabolic process and immune responses (Figure 5B). These findings were validated by qRT-PCR for targets including *IL1B*, *IL36G*, C-X-C motif chemokine ligand 1 (*CXCL1*), *CCL20*, and epidermal barrier and antimicrobial genes such as *DEFB4*, *S100A7*, *FLG*, *LOR*, and *IVL* (Figure 5C). Consistent with these findings, pretreatment of keratinocytes with a cPLA2 inhibitor (AACOCF3) or sPLA2 inhibitor (LY333013 and S3319) suppressed induction of proinflammatory genes (*IL1B*, *CXCL1*, and *CCL20*) and skin barrier genes (*S100A7* and *IVL*) by TNF plus IL-17A stimulation (Supplemental Figure 7, A and B).

Taken together, these data demonstrate that the 3 PLA2 enzymes act downstream of TNF- and IL-17A–driven inflammatory responses and modulate epidermal homeostasis.

### PLA2s regulate specific phospholipid/eicosanoid species that contribute to immune responses and skin barrier function.

**Figure 6:**
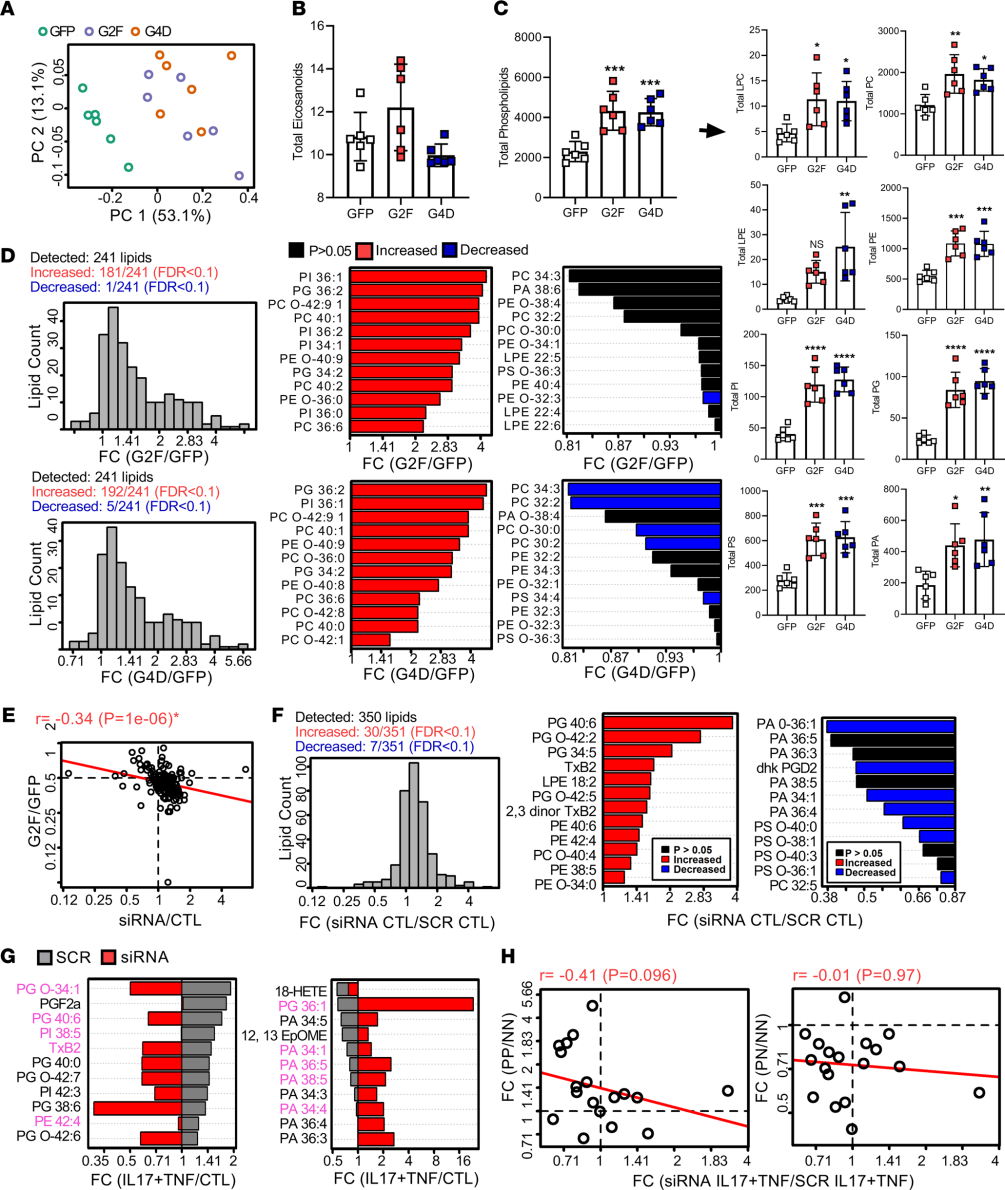
PLA2s modulate phospholipid/eicosanoid profiles in keratinocytes. (**A**) Principal component analysis performed based on the abundance of 266 phospholipid and eicosanoids in PLA2G2F- or PLA2G4D-overexpressing keratinocytes. (**B**) The average total eicosanoid abundance (± 1 SEM) in each group (*n* = 6/group). Data are expressed as pmol/mL supernatant. Groups without the same letter differ significantly from one another (*P* < 0.05; Fisher’s least significant difference). (**C**) The average total phospholipid abundance (± 1 SEM) in each group averaging across all phospholipid categories (LPC + PC + LPE + PE + LPI + PI + LPG + PG + LPS + PS + LPS + PA) (*n* = 6/group). LPC, lyso-phosphatidylcholine; PC, phosphatidylcholine; LPE, lyso-phosphatidylethanolamine; PE, phosphatidylethanolamine; PI, phosphatidylinositol; PG, phosphatidylglycerol; PS, phosphatidylserine; PA, phosphatidic acid. Data are expressed as normalized intensities and constitute relative abundances per 1 × 10^6^ cells (relative to internal standards). (**D**) Phospholipids most strongly altered by PLA2G2F and PLA2G4D overexpression. (**E**) Changes in eicosanoid abundance following PLA2G2F siRNA knockdown and correlation with changes observed in PLA2G2F-overexpressing keratinocytes. (**F**) Lipids with most strongly altered abundance following PLA2G2F siRNA knockdown in keratinocytes. (**G**) Lipids with the most siRNA-dependent IL-17A and TNF responses. (**H**) Changes in eicosanoid abundance following PLA2G2F siRNA knockdown and correlation with changes observed in lesional (psoriasis, PP), uninvolved (PN), and normal (NN) skin biopsies. *P < 0.05, **P < 0.01, ***P < 0.001, ****P < 0.0001 by 1-way ANOVA (**B** and **C**) or Pearson’s correlation coefficient (**H**).

The PLA2 family is a major regulator of lipid metabolism (20). To determine the immune regulatory roles of PLA2-regulated lipids in psoriasis, we first performed liquid or gas chromatography–mass spectrometry (LC/GC-MS) analysis of phospholipids and eicosanoids in normal (NC), nonlesional, and psoriatic skin (PV). As the distribution of FC estimates (PV/NC) showed, 98 phospholipid metabolites were increased, and 2 decreased in psoriatic lesions (*n* = 10) (Supplemental Figure 8A), but none were significantly changed in nonlesional skin (*n* = 10) (Supplemental Figure 8B) compared to normal controls (*n* = 10). The phospholipid profile in psoriatic skin was characterized by higher concentrations of phosphatidylcholines (PCs), including 30:2, 32:0, and 30:1 PC, and lower concentrations of phosphatidylethanolamines (PEs), including 42:0 and 42:1 PE (Supplemental Figure 8A). Compared with normal control skin, 45 eicosanoid metabolites were significantly increased, 1 decreased in psoriatic lesions (Supplemental Figure 8C), and none were significantly changed in nonlesional skin (*n* = 10) (Supplemental Figure 8D). The most abundant eicosanoids detected in the psoriatic lesions were α-linolenic acid (ALA), docosahexaenoic acid (DHA), eicosapentaenoic acid (EPA), and derivatives of linoleic acid (LA) (13-HOTrE), arachidonic acid (AA) (8- and 9-HETE), and oxidized-DHA products (11-, 13-, 7-, 10-, 8-, 20-HDoHE) (Supplemental Figure 8C), corresponding to the same phospholipids and eicosanoids seen in our in vitro experiments (Figure 6 and Supplemental Table 3). The only significantly decreased eicosanoid was resolvin D1 (FDR < 0.1), an antiinflammatory mediator (21) derived from omega-3 polyunsaturated fatty acids (Supplemental Figure 8C).

Next, we assessed phospholipids and eicosanoids by GC/LC-MS in OE and siRNA-treated keratinocytes for the sPLA2, *PLA2G2F*, and the cPLA2, *PLA2G4D*. PCA using the abundance of the 266 phospholipids and eicosanoids showed complete separation of the control samples from both *PLA2G2F*- and *PLA2G4D*-OE keratinocytes (Figure 6A). Total eicosanoid abundance was slightly higher in the *PLA2G2F*-OE keratinocytes compared with control, whereas it was slightly lower in *PLA2G4D*-OE keratinocytes, although the difference was not significant (Figure 6B). In contrast, total phospholipid abundance was significantly increased in both *PLA2G2F*-OE (1.88-fold, *P* = 0.000493) and *PLA2G4D*-OE (1.87-fold, *P* = 0.000223) keratinocytes compared with control (Figure 6C). In addition, among the 241 individual phospholipid species detected, 181 were increased in *PLA2G2F*-OE keratinocytes, and only 1 was decreased (FDR < 0.10; Figure 6D). Likewise, 192 phospholipid species were increased in *PLA2G4D*-OE keratinocytes, and 5 were decreased (FDR < 0.10; Figure 6D). The most strongly increased phospholipids were phosphatidylinositol (PI) 36:1, phosphatidylglycerol (PG) 36:2, and PC O-42:9 in both the *PLA2G2F*- and *PLA2G4D*-OE groups (Figure 6D). Changes in individual lipid species are shown in Supplemental Table 3.

We also performed LC-MS lipidomics analysis in *PLA2G2F*-silenced keratinocytes with or without TNF plus IL-17A treatment. There was a significant negative correlation between *PLA2G2F*-OE versus GFP control, and *PLA2G2F*-silenced versus control estimates (*P* = 0.025; Figure 6E). This indicated that changes in eicosanoid abundance resulting from *PLA2G2F* OE were opposite to those observed with *PLA2G2F* silencing. Moreover, 351 lipids were differentially secreted by *PLA2G2F*-silenced keratinocytes compared with controls, leading to substantial increases in 30 lipids, including PG 40:6, PG 42:9, PG 34:5, thromboxane B2 (TXB2), and decreases in 7 lipids, including phosphatidic acid (PA) O-36:1 (FDR < 0.10; Figure 6F). Further, TNF plus IL-17A rapidly and transiently modulated lipid metabolism in keratinocytes (Supplemental Figure 8E and Figure 6G), which was strongly reversed by *PLA2G2F* silencing (Figure 6G). Furthermore, the lipid abundance changes in TNF- plus IL-17A–treated *PLA2G2F*-silenced keratinocytes (Supplemental Figure 8F) correlated negatively with changes observed in lesional (*r* = –0.41, *P* = 0.096) but not with nonlesional psoriatic skin (*r* = –0.01, *P* = 0.97) (Figure 6H), suggesting that phospholipid/eicosanoid profiling in psoriatic lesions was most strongly influenced by PLA2G2F. 

To determine if PLA2-regulated lipids directly affected the expression of proinflammatory genes in psoriasis, we stimulated keratinocytes in vitro with PC and DHA. This markedly increased the expression of proinflammatory genes, including *IL1B*, *IL36G*, *CXCL1*, *CCL20*, and *S100A7*, and epidermal barrier genes such as *IVL* and *FLG* (Supplemental Figure 9). These results suggest that PLA2s, especially PLA2G2F, regulate immune responses and keratinocyte differentiation through modulation of lipid species in the epidermis.

### PLA2 inhibition alleviates psoriasis-like inflammation in vivo.

**Figure 7:**
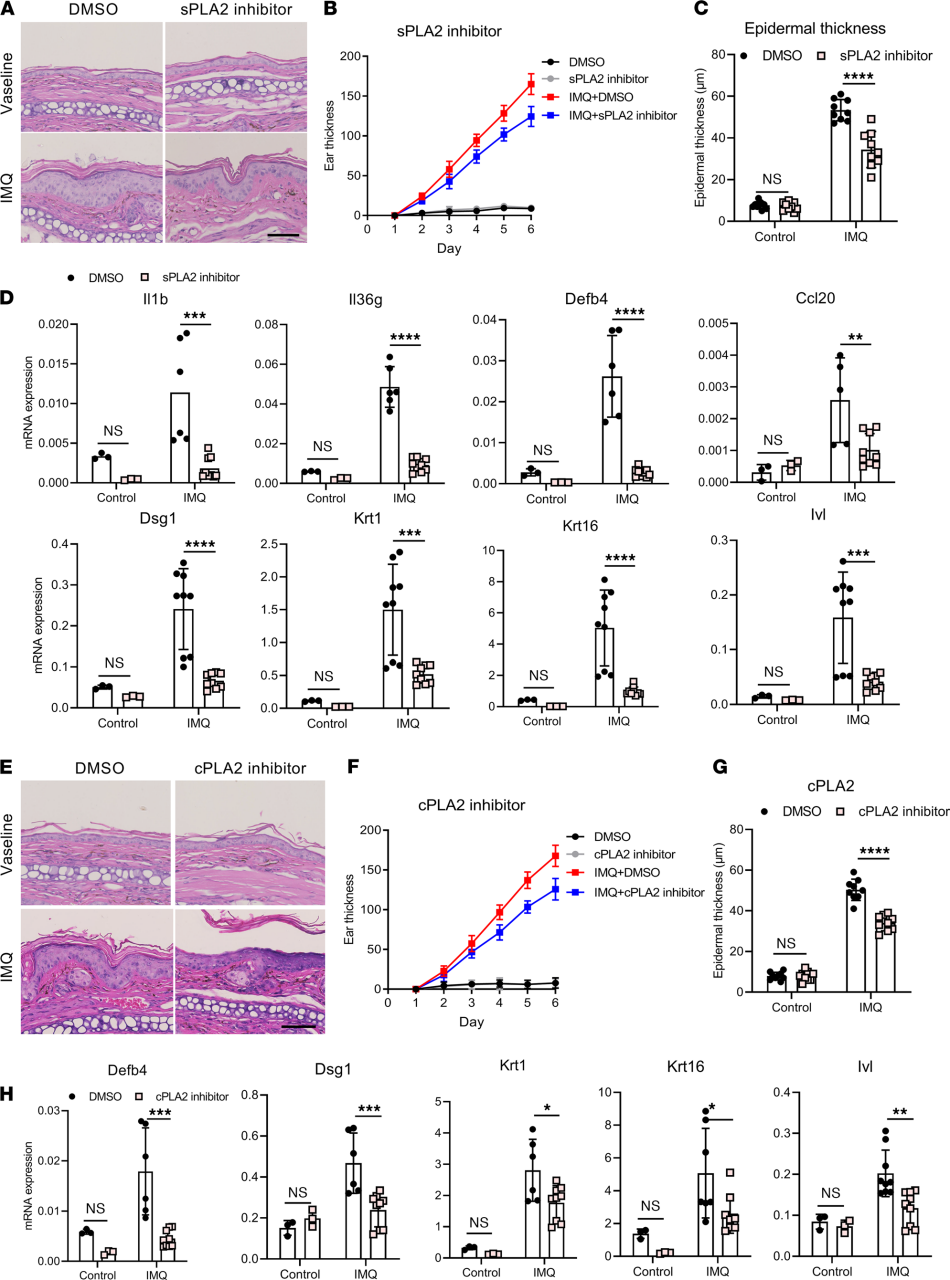
PLA2 inhibition alleviates psoriasis-like inflammation in vivo. Chemical inhibitors targeting sPLA2 and cPLA2 were topically applied to IMQ-induced psoriasis-like mice (*n* = 5) to block PLA2 function. (**A**) H&E staining of ear sections from IMQ-induced mice with sPLA2 inhibitor treatment on day 6. Scale bar: 50 μm. (**B**) Dynamic changes in ear thickness at the indicated time points. (**C**) Epidermal thickness was evaluated based on the data in **A**. (**D**) qRT-PCR results showing mRNA expression of various cytokines and differentiation-related genes in the ears of IMQ mice on day 6. (**E**) H&E staining of ear sections from IMQ-induced mice with sPLA2 inhibitor treatment on day 6. Scale bar: 50 μm. (**F**) Dynamic changes in ear thickness at the indicated time points. (**G**) Epidermal thickness was evaluated based on the data in **E**. (**H**) qRT-PCR results showing mRNA expression of *Defb4* and differentiation-related genes in the ears of each indicated group on day 6. Two-way ANOVA. Data are presented as mean ± SEM. *P < 0.05, **P < 0.01, ***P < 0.001, ****P < 0.0001.

To validate the role of PLA2s in vivo, we used the IMQ-induced psoriasis-like model and applied topical inhibitors of sPLA2 and cPLA2 in 2 independent experiments. Application of a topical sPLA2 inhibitor over a 5-day period resulted in decreased ear thickness, epidermal thickness, and inflammatory infiltrates (Figure 7, A–C). qRT-PCR analysis of inflamed skin from IMQ mice treated with the sPLA2 inhibitor revealed significantly lower mRNA expression of proinflammatory genes (*Il1b*, *Il36g*, *Defb4*, and *Ccl20*) and differentiation-related genes (*Dsg1*, *Krt1*, *Krt16*, and *Ivl*), in addition to suppression of *Pla2g2f* expression itself (Supplemental Figure 10) compared with control-treated mice (Figure 7D). Furthermore, similar responses were seen in cPLA2 inhibitor–treated IMQ mice, including decreased ear thickness, acanthosis, and inflammatory cell infiltrates (Figure 7, E–H). However, the impact on gene expression was more modest in cPLA2 inhibitor–treated IMQ mice compared with sPLA2 inhibitor treatment, and was limited to differentiation and antimicrobial genes, including *Defb4*, *Dsg1*, *Krt1*, *Krt16*, and *Ivl* (Figure 7H). These data suggest that targeting PLA2s in psoriasis may have a therapeutic effect and that targeting sPLA2 has a greater antiinflammatory impact.

## Discussion

Psoriasis and PRP share many clinical and histologic features, including marked epidermal hyperproliferation, abnormal differentiation, and inflammatory responses. However, while multiple highly effective treatments exist for psoriasis, PRP generally tends to be treatment resistant (22), suggesting different pathologic mechanisms, which may be downstream of proinflammatory cytokines known to be key drivers of psoriasis pathogenesis (23). To determine if PRP and psoriasis share pathogenic mechanisms, we performed a transcriptomic study using archived formalin-fixed, paraffin-embedded (FFPE) biopsy samples from a cohort of patients with PRP along with available FFPE samples from previous psoriasis studies (24–26) for comparison. This led to our identification of 3 members of the PLA2 family, both soluble (PLA2G2F) and cytoplasmic (PLA2G4D and PLA2G4E), as a critical pathogenic pathway shared between PRP and psoriasis. Our data further suggest that these enzymes are critical components downstream of TNF and IL-17A, a major circuit in psoriasis pathogenesis, and are sufficient to promote abnormal differentiation and expression of additional proinflammatory cytokines, thereby providing a plausible pathway by which they may promote many of the changes in both psoriasis and PRP.

The PLA2G2F, PLA2G4D, and PLA2G4E enzymes are part of the PLA2 family, which has over 30 members, consisting of both cPLA2s (such as PLA2G4D and PLA2G4E), and sPLA2s (i.e., PLA2G2F), with variable expression of different members in a broad range of tissues (11, 20). Studies have demonstrated that PLA2 enzymes regulate lipid pathways, and that many of these lipid species generated by these enzymes serve as bioactive mediators and essential components of the skin barrier (20, 27). In addition, individual PLA2 enzymes exhibit unique tissue and cellular distribution, suggesting that they have distinct biological roles in production of pro- and antiinflammatory lipid mediators (20, 28). In psoriasis and PRP skin the 3 PLA2s are primarily expressed in the epidermis, with minimal contribution from other cell types. Of the 3 upregulated PLA2s that we identified, PLA2G2F is the best studied in skin homeostasis, and is expressed in the suprabasal epidermis where it is upregulated during calcium-induced differentiation or stimulation with IL-22 (29). It has been shown to play a fundamental role in hyperproliferative epithelial diseases such as psoriasis and skin cancers (29, 30). PLA2G4D, a cPLA2, has been demonstrated to be expressed in psoriatic mast cells and promote release of exosomes, and contributes to generation of neolipid skin antigens presented to CD1a-reactive T cells, leading to the production of IL-22 and IL-17A (10). Our data demonstrate that the combination of TNF and IL-17A is a strong inducer of PLA2G2F, PLA2G4D, and PLA2G4E expression, and that these PLA2s play a major role in the proinflammatory effects of IL-17A and TNF on the epidermis, as pharmacologic inhibition or siRNA-mediated silencing of these enzymes leads to marked blunting of TNF and IL-17A responses in both inflammatory and differentiation-related processes. Furthermore, if the expression of these enzymes is autonomous of IL-17A and TNF, it may provide an explanation for the lack of treatment response seen in up to 50% of patients with PRP to anti-TNF, anti–IL-17A, or anti–IL-23 agents (22, 31).

Our lipid profiling demonstrates marked changes in phospholipid and eicosanoid synthesis in psoriatic skin. This includes abundant arachidonic acid metabolites, DHA and oxidized-DHA products, and PCs, and decreased PE, LPC, and resolvin D1, and are consistent with previous findings (32, 33). Lipid mediators can serve as both activators and suppressors of inflammation to elicit local effects (34). For example, leukotriene B4 (LTB4) (35) and 12-HETE (35) can act as chemoattractants for neutrophils and macrophages in the skin. In contrast, 15-HETE may act as a negative regulator in LTB4- and 12-HETE–induced inflammation (36). DHA, a PLA2 metabolite, can affect skin homeostasis via activating keratinocytes to express proinflammatory mediators. In addition, resolvin D1, a DHA-derived antiinflammatory eicosanoid that is decreased in psoriatic skin and downregulated by PLA2s, exerts a protective role in psoriasis-like dermatitis and other types of inflammatory responses (21, 34, 37). Therefore, we consider that the imbalance between proinflammatory and antiinflammatory lipid mediators modulated by these 3 PLA2s may shift the balance in skin toward an environment that promotes proinflammatory responses characteristic of both psoriasis and PRP.

Furthermore, PLA2s can signal through some proinflammatory pathways to regulate immune responses. Sphingolipid metabolism in astrocytes has been reported to trigger cPLA2 recruitment to mitochondrial antiviral signaling protein (MAVS), boosting NF-κB–driven transcriptional programs that promote inflammation in experimental autoimmune encephalomyelitis (28). Moreover, sPLA2 enhances Toll-like receptor activity, as it can increase the intracellular uptake of poly(I:C), inducing phosphorylation of NF-κB and mitogen-activated protein kinases (MAPKs) in keratinocytes (38). Another PLA2, sPLA2-IIA, regulates the proliferation of tissue stem cells and cancer cells through common JNK/c-Jun signaling, thus contributing to tissue homeostasis and repair (39). Therefore, the mechanism underlying PLA2s’ regulation of the immune response in keratinocytes warrants further investigations.

Finally, we demonstrate that targeting sPLA2 or cPLA2 may be a novel approach for treatment of psoriasis and other inflammatory skin diseases characterized by epidermal hyperplasia and epidermal inflammatory responses. Inhibitors of PLA2 have been actively studied and are under development (40), suggesting that deeper knowledge of the biology of these enzymes and their disease context is needed. For example, the cPLA2α inhibitor AVX001 has shown efficacy and favorable safety profile as a topical agent for the treatment of plaque psoriasis in a randomized, double-blind, placebo-controlled phase I/IIa clinical trial (41). Further study demonstrates that AVX001 exerts antiinflammatory and antiproliferative effects on peripheral blood mononuclear cells and HaCaT cells (immortalized human keratinocytes), probably through reduced local eicosanoid availability (42). Moreover, another cPLA2 inhibitor, AACOCF3, has been reported to reduce IL-1β–induced eicosanoid production (43). BMS-229724, a selective inhibitor of cPLA2, alleviates phorbol ester–induced chronic inflammation, including edema and neutrophil infiltration, when applied topically on mouse ears (44). Additionally, in a mouse model of asthma, AACOCF3 prevents development of airway hyperresponsiveness (45). For sPLA2 inhibitors, one study has explored the possibility of using sPLA2 inhibitor–loaded micelles to treat neuropathic pain (46). Thus, inhibitors of sPLA2 or cPLA2 have robust antiinflammatory effects and their potential for treatment of psoriasis, and PRP, should be further explored in future studies.

A limitation of our study is a lack of lipidomic analysis of PRP skin to compare against psoriatic skin and is mainly due to the rarity of PRP and difficulty in recruiting a prospective cohort of patients. An additional confounding variable is the heterogeneity of PRP, which currently includes 6 different clinical subtypes (3).

In summary, our study reveals a previously unrecognized role of PLA2G2F, an epidermal sPLA2, and PLA2G4D and PLA2G4E, epidermal cPLA2s, in skin homeostasis and inflammation, and through our in vitro and in vivo models demonstrates and highlights the potential promise of targeting PLA2s in psoriasis and PRP.

## Methods

### Microarray analysis.

Biopsies of psoriasis (*n* = 22) or PRP (*n* = 13, type 1) cases were identified through the University of Michigan Pathology database. The details of patient sample information are provided in Supplemental Table 4. Control blocks were obtained from healthy volunteers. RNA was isolated from 10-μm sections of FFPE blocks of identified skin biopsies using the E.N.Z.A. FFPE RNA Kit (Omega Bio-tek). Experimental details and data analysis were done as we previous published (47).

### RNA-seq and analysis.

RNA was isolated from cell cultures using the RNeasy plus kit (Qiagen). All samples had an RNA integrity number (RIN) above 9.5, as assessed by the Agilent Bioanalyzer. Libraries for RNA-seq were generated from polyadenylated RNA and sequenced at 6 libraries per lane on the Illumina Genome Analyzer IIx. Tophat2 was used to align RNA-seq reads to the human genome, using annotations of GENCODE as the gene model. HTSeq was used to quantify gene expression levels. Normalization and differential expression analysis were performed by DESeq2. Differential gene expression analysis was performed with the functions glmQLFit and glmQLFTest (R package: edgeR), which reports the *P* value, FDR, and log_2_FC for pair-wise comparison and gene. PCA was done on normalized and sample-averaged gene expression data for each array. GO analysis of RNA-seq data was performed using DAVID (Database for Annotation, Visualization and Integrated Discovery; http://david.abcc.ncifcrf.gov).

### scRNA-seq and data analysis.

For the generation of single-cell gel beads in emulsion, cells were loaded on a Chromium single-cell instrument (10× Genomics) with an estimated targeted cell recovery of approximately 5,000 cells, as per the manufacturer’s protocol. scRNA-seq libraries were prepared by using the Chromium single-cell 3′ library and gel bead kit v3 (10× Genomics). Sequencing was performed using the Illumina NovaSeq, and the digital expression matrix was generated using the Cell Ranger pipeline (10× Genomics).

To identify different cell types and find signature genes for each cell type, the R package Seurat (version 3.0.2) was used to analyze the digital expression matrix. Cells with fewer than 500 unique molecular identifiers (UMIs) and greater than 25% mitochondrial expression were removed from further analysis. The Seurat function NormalizeData was used to normalize raw counts and variable genes were identified using the FindVariableGenes function. The Seurat ScaleData function was used to scale and center expression values in the data set for dimensional reduction. PCA, t-distributed stochastic neighbor embedding (t-SNE), and uniform manifold approximation and projection (UMAP) were used for dimensionality reduction, and the first 2 dimensions were used in plots. The FindClusters function was later used to cluster the cells. Signature genes were then found and used to define cell types for each cluster. Keratinocytes were selected based on high expression of the *KRT5* and *KRT10* genes.

### Lipidomics of skin, cells, and supernatants.

Lipid analysis was performed at the UCSD Lipidomic Core as described previously (48) for each lipid species.

### 3D human skin equivalents.

3D human skin equivalents were established as previously described using primary neonatal foreskin–derived keratinocytes (49). Skin models were fixed with 10% neutral buffered formalin overnight, embedded in paraffin wax, and cut into 4 μm thickness. Sections were subsequently stained with H&E to assess general morphology.

### Mouse experiments.

Female C57BL/6J mice (8–12 weeks old) used in the current study were purchased from the Department of Laboratory Animal Medicine of the Fourth Military Medical University. Mice were randomly assigned to groups of 5 mice and maintained in a specific pathogen–free barrier facility.

The IMQ-induced psoriasis model was prepared by topically applying 5 mg of Aldara cream (containing 5% IMQ, INova Pharmaceuticals) or control cream on mouse ears for 5 consecutive days. To investigate the effects of PLA2 on psoriasis, the inhibitors targeting PLA2 (20 μg/ear/d) were topically applied on the mouse ear every day. Then, skin specimens were obtained from mice for further analysis. The following inhibitors were used: varespladib methyl (HY-17448, MedChemExpress) and CAY10650 (HY-10801, MedChemExpress).

### Primary keratinocyte culture and treatment.

Normal human keratinocytes were established from healthy adults, as previously described (50). Skin biopsies were obtained from volunteer patients following protocols approved by the University of Michigan institutional review board. Informed consent was obtained from all subjects. Cultures were maintained with serum-free medium (Medium 154, Invitrogen/Cascade Biologics) and used at passage 2 or 3 with a calcium concentration of 0.1 mM. Then cells were starved of growth factors for 24 hours before treatment with recombinant human IL-4 (10 ng/mL, 204-IL-010, R&D Systems), IL-13 (10 ng/mL, 213-ILB-005, R&D Systems), IFN-α (20 ng/mL, I4276, Sigma-Aldrich), IFN-γ (10 ng/mL, 285-IF, R&D Systems), TNF (10 ng/mL, 210-TA-020, R&D Systems), IL-17A (10 ng/mL, 317-ILB-050, R&D Systems), TNF plus IL-17A, prostaglandin E2 (10 μM, P0409, Sigma-Aldrich), PC (10 μM, HY-B2233B, MedChemExpress), and DHA (10 μM, HY-B2167, MedChemExpress). PLA2 inhibitors were used in cell experiments to block the functions of PLA2s, including sPLA2 inhibitors LY333013 (10 μM, HY-17448, MedChemExpress) and S3319 (10 μM, Sigma-Aldrich), and cPLA2 inhibitor AACOCF3 (10 μM, A231, Sigma-Aldrich). After 24 hours of stimulation, RNA was extracted by RLT buffer for subsequent RNA-seq and qRT-PCR experiments.

Protocols for siRNA experiments, overexpression of PLA2G2F and PLA2G4D, qRT-PCR, Western blots, and immunofluorescence microscopy are detailed in supplemental materials and methods.

### Data and materials availability.

All data are available in the main text or the supplementary materials. The normalized and raw data files are available in the NCBI’s Gene Expression Omnibus database (GEO GSE183134).

### Statistics.

Calculations were made using GraphPad Prism version 9. For in vitro studies, statistical significance was determined using unpaired, 2-tailed Student’s t test or analysis of variance (ANOVA) as indicated in the legends. For RNA-seq analysis, FDR was used to control for multiple testing. A nominal *P* value of less than 0.05 was considered significant for all statistical tests.

### Study approval.

Human samples were obtained from volunteer psoriasis, PRP, AD patients, and healthy controls with protocols approved by the University of Michigan institutional review board. Informed consent was obtained from all subjects prior to inclusion in the study in accordance with Declaration of Helsinki principles. Participants were identified by number, not by name. All mouse experiments were performed in compliance with the NIH *Guide for the Care and Use of Laboratory Animals* (National Academies Press, 2011) and were approved by the University of Michigan Review Committee for the Use of Animals.

## Author contributions

SS, JC, XX, RU, MKS, and OP conducted experiments, processed samples, and interpreted the data. WRS, LCT, FM, and RW performed bioinformatics analyses. SS, JEG, GW, ACB, KMS, PH, VES, EM, JMK, NLW, and PWH contributed to the design of the study and preparing, writing, and revising of the manuscript.

## Supplementary Material

Supplemental data

Supplemental table 1

Supplemental table 2

Supplemental table 3

Supplemental table 4

## Version 1 09/07/2021

In-Press Preview

## Version 2 10/22/2021

Electronic publication
