# Supplementary material for: Phospholipase A2 enzymes represent a shared pathogenic pathway in psoriasis and pityriasis rubra pilaris
Source: JCI Insight. 2021 Oct 22;6(20):e151911. doi: 10.1172/jci.insight.151911 (PMC8564909; doi:10.1172/jci.insight.151911)
Supplement: Supplemental data [file jciinsight-6-151911-s008.pdf]

Supplementary Materials for

Phospholipase A2 enzymes represent a shared pathogenic pathway in psoriasis and pityriasis rubra pilaris

Shuai Shao[1,2,*], Jiaoling Chen[1], William R. Swindell[2,3], Lam C. Tsoi[2], Xianying Xing[2], Feiyang Ma[4], Ranjitha Uppala[2], Mrinal K. Sarkar[2], Olesya Plazyo[2], Allison C. Billi[2], Rachael Wasikowski[2], Kathleen M. Smith[5], Prisca Honore[6], Victoria E. Scott[6], Emanual Maverakis[7], J. Michelle Kahlenberg[8], Gang Wang[1], Nicole L. Ward[9], Paul W. Harms[2,10], Johann E. Gudjonsson[2,*]

Affiliations:

[1]Department of Dermatology, Xijing hospital, Fourth Military Medical University, Xi'an, Shannxi, 710032, China.

[2]Department of Dermatology, University of Michigan, Ann Arbor, Michigan, MI, 48109, USA.

[3]Department of Internal Medicine, the Jewish Hospital, Cincinnati, OH, 45236, USA.

[4]Department of Dermatology, Department of Medicine, University of California, Los Angeles, CA, 90095, USA.

[5]Abbvie Cambridge Research Center, Cambridge, Massachusettes, MA, 02138, USA.

[6]AbbVie Dermatology Discovery, North Chicago, IL 60064, USA.

[7]Department of Dermatology, University of California Davis School of Medicine, Sacramento, California, 95616, USA.

[8]Division of Rheumatology, Department of Internal Medicine, University of Michigan, Ann Arbor, Michigan, 48109, USA.

[9]Departments of Nutrition and Dermatology, Case Western Reserve University, Cleveland, Ohio, 44106, USA.

[10]Department of Pathology, University of Michigan Medical School, Ann Arbor, Michigan, 48109, USA.

[†]Address for correspondence: Dr. Johann E. Gudjonsson, Department of Dermatology, University of Michigan, Ann Arbor, Michigan, 48109, USA; johanng@med.umich.edu; or Dr. Shuai Shao, Department of Dermatology, Xijing Hospital, Fourth Military Medical University, Xi'an, Shannxi, 710032, China; and Department of Dermatology, University of Michigan, Ann Arbor, Michigan, 48109, USA; 857746654@qq.com.

Conflict of interest statement: The authors have declared that no conflict of interest exists.

This PDF file includes:

Supplementary materials and methods

Supplementary figures and figure legends:

Figure S1. The expression level of PLA2G2F and PLA2G4D in several skin diseases.

Figure S2. ScRNA-seq analysis of psoriatic lesional skin and controls for three PLA2 genes.

Figure S3. The expression level of PLA2G2F, PLA2G4D, and PLA2G4E in 3D human skin equivalents.

Figure S4. The expression level of PLA2G2F, PLA2G4D, and PLA2G4E in cytokine-treated keratinocytes.

Figure S5. Overexpressing PLA2G2F or PLA2G4D modulates the immune response and skin barrier genes in keratinocytes.

Figure S6. The knockdown efficiency of PLA2G2F, PLA2G4D, and PLA2G4E in keratinocytes.

Figure S7. Pharmacological inhibiting sPLA2 or cPLA2 alleviates inflammatory responses and helps normalize differentiation *in vitro*.

Figure S8. Lipids with most strongly altered abundance in comparisons among PP and NC skin samples, and in IL-17A+TNF stimulated keratinocytes.

Figure S9. The proinflammatory role of some PLA2s-regulated lipids.

Figure S10. Inhibitors of PLA2s reduce the mRNA expression level of Pla2s in IMQ-induced mice.

Supplementary materials and methods

siRNA experiments

Small interfering RNA (siRNA) targeting PLA2s including PLA2G2F (Accell Human PLA2 siRNA, E-008591-00-0005), PLA2G4D (E-008599-00-0005), and PLA2G4E (E-010297-00-0005), and control siRNA (D-001910-01-20) were purchased from Dharmacon company and introduced into cells by Delivery medium (B-005000-100, Dharmacon) according to the manufacturer's instructions.

Overexpression of PLA2G2F and PLA2G4D

N/TERT keratinocytes stably overexpressing PLA2s were generated using 4D-Nucleofector X Unit (Lonza Cologne). Cells were prepared using a standard protocol for the Normal Human Epidermal Keratinocyte X Unit kit (4D Nucleofector Solution, supplement and 100μL single nucleocuvette) obtained from Lonza. For each electroporation, 5μg pCMV6-AC-GFP PLA2G2F (RG220309, Origene) or PLA2G4D (RG222969, Origene) plasmid was used. Unit X program DS-138 was selected for stable keratinocytes. Following transfection, keratinocytes were grown in a 12-well plate using fully supplemented Keratinocyte-SFM medium, penicillin streptomycin, and 500μg/mL G418 (Thermo Fisher Scientific) for selection followed by expansion for approximately 30 days. PLA2 overexpression was validated using western blotting and qRT-PCR (Figure S5A).

QRT-PCR

Total RNA was isolated using RNeasy plus kit (74136, Qiagen) and RNA yield was assessed using a spectrophotometer (Epoch™ Microplate Spectrophotometer, BioTek, Winooski, VT). 1 μg of total RNA was reverse transcribed using the High Capacity cDNA Reverse Transcription Kit (Life Technology) following the manufacturer's instructions. TaqMan gene expression assays (Thermo Fisher Scientific) were used to assess relative expression of selected genes and normalized to RPLP0. Then qRT-PCR was performed on an ABI PRISM 7900 Sequence Detection HT system (Applied Biosystems). The method of 2^ Ct was used to obtain the relative mRNA expression of genes of interest by normalizing their Ct values with

Ct values of the reference gene RPLP0. Primers (Thermo Fisher Scientific) used in this study were: PLA2G2F, Hs00224482_m1; PLA2G4D, Hs00603557_m1; PLA2G4E, Hs00416278_m1; IL1B, Hs01555410_m1; IL36G, Hs00219742_m1; S100A7, Hs01923188_u1; DEFB4, Hs00175474_m1; CCL20, Hs00355476_m1; IVL, Hs00846307_s1; FLG, Hs00856927_g1; LOR, Hs01894962_s1; CXCL1, Hs00236937_m1; RPLP0, Hs00420895_gH.

Western blots

Cells were washed with PBS and isolated using Pierce RIPA buffer (89900, Thermo Fisher Scientific) containing PSMA (Sigma) and 1×protease inhibitor cocktail (Roche). Afterwards, samples were run on a precast gel (456-1094S, Bio-Rad) and transferred to a PVDF membrane. The following antibodies were used for Western blot analysis: anti-PLA2G2F (sc-58363, Santa Cruz Biotechnology), anti-PLA2G4D (PA5-72287, Invitrogen), and anti-β-actin (A5441, Sigma); followed by secondary antibodies (anti-mouse or rabbit IgG, AP-linked Antibody, Cell Signaling). Blots were then washed 3 times and substrate was added (45-000-947, Fisher Scientific). The membrane was scanned using the Molecular Dynamics STORM 860 PhosphorImager (GE Health Care, STORM 860)

Immunofluorescence microscopy

Paraffin-embedded skin sections were dewaxed using Citroclear, rehydrated in a series of descending gradient of ethanol- water solutions, and then boiled in 1× Target Retrieval buffer (Dako) for 15 minutes for antigen retrieval. Sections were then cooled down in PBS and blocking was done with 1% BSA in PBS for 1 hour at room temperature. Skin sections were then ready for incubation with primary and secondary antibodies, accordingly. Coverslips were mounted in Fluoroshield mounting medium (Sigma-Aldrich). For LAD2 mast cell imaging, cells were plated on culture slides (BD) pretreated with poly-d-lysine, fixed and permeabilized with acetone for 10 minutes, and then blocked with blocking solution for 1 hour at room temperature, followed by staining with primary and secondary antibodies. Primary antibodies were: rabbit anti-PLA2G2F (5ug/ml, sc-164549, Santa Cruz

Biotechnology), rabbit anti-PLA2G4D (10/ml, LS-C119894/68125, Lifespan biosciences. Secondary antibodies were: goat anti-rabbit IgG-Alexa Fluor 488 (1:500; Thermo Fisher Scientific). Images were acquired on an Axiovert S100 microscope (ZEISS) coupled with a digital camera (ORCA-ER C4742-80; Hamamatsu Photonics). Images were processed by ZEN imaging software (Blue edition; ZEISS).

Supplementary figures and figure legends

Figure S1

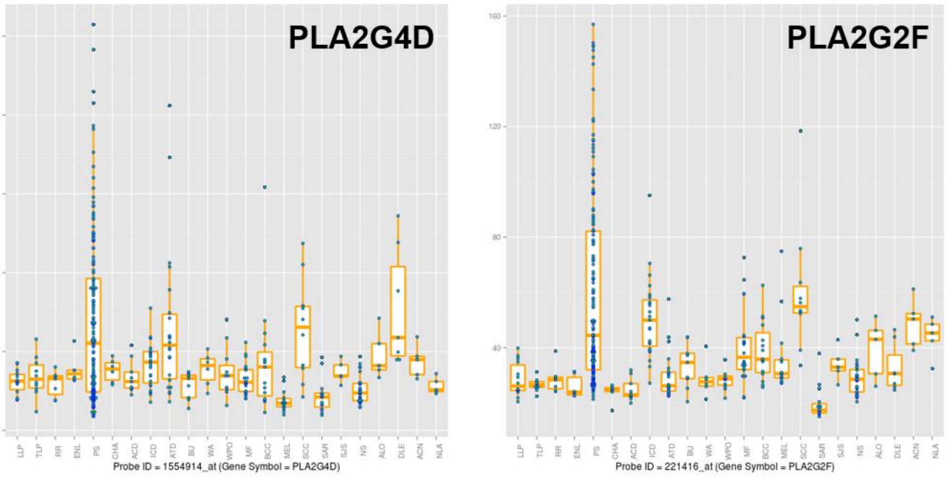

ACD: Allergic contact dermatitis
ACN: Acne
ALO: Alopecia areata
ATD: Atopic dermatitis
BCC: Basal cell carcinoma
BU: Burn
CHA: Chancroid
DLE: Discoid Lupus Erythematosus
ENL: Erythema nodosum leprosum
ICD: Irritant contact dermatitis
LLP: Lepromatous leprosy
MEL: Melanoma
MF: Mycosis fungoides
NLA: Non-lesional Acne
NS: Normal skin
PS: Psoriasis
RR: Reversal reaction (leprosy)
SAR: Cutaneous sarcoidosis
SCC: Squamous cell carcinoma
SJS: Stevens Johnson syndrome (blister cells)
TLP: Tuberculoid leprosy
WA: Acute wound (0h after injury)
WPO: Post-operative wound (3, 7 days after injury)

Figure S1. The expression level of PLA2G2F and PLA2G4D in several skin diseases.

Figure S2

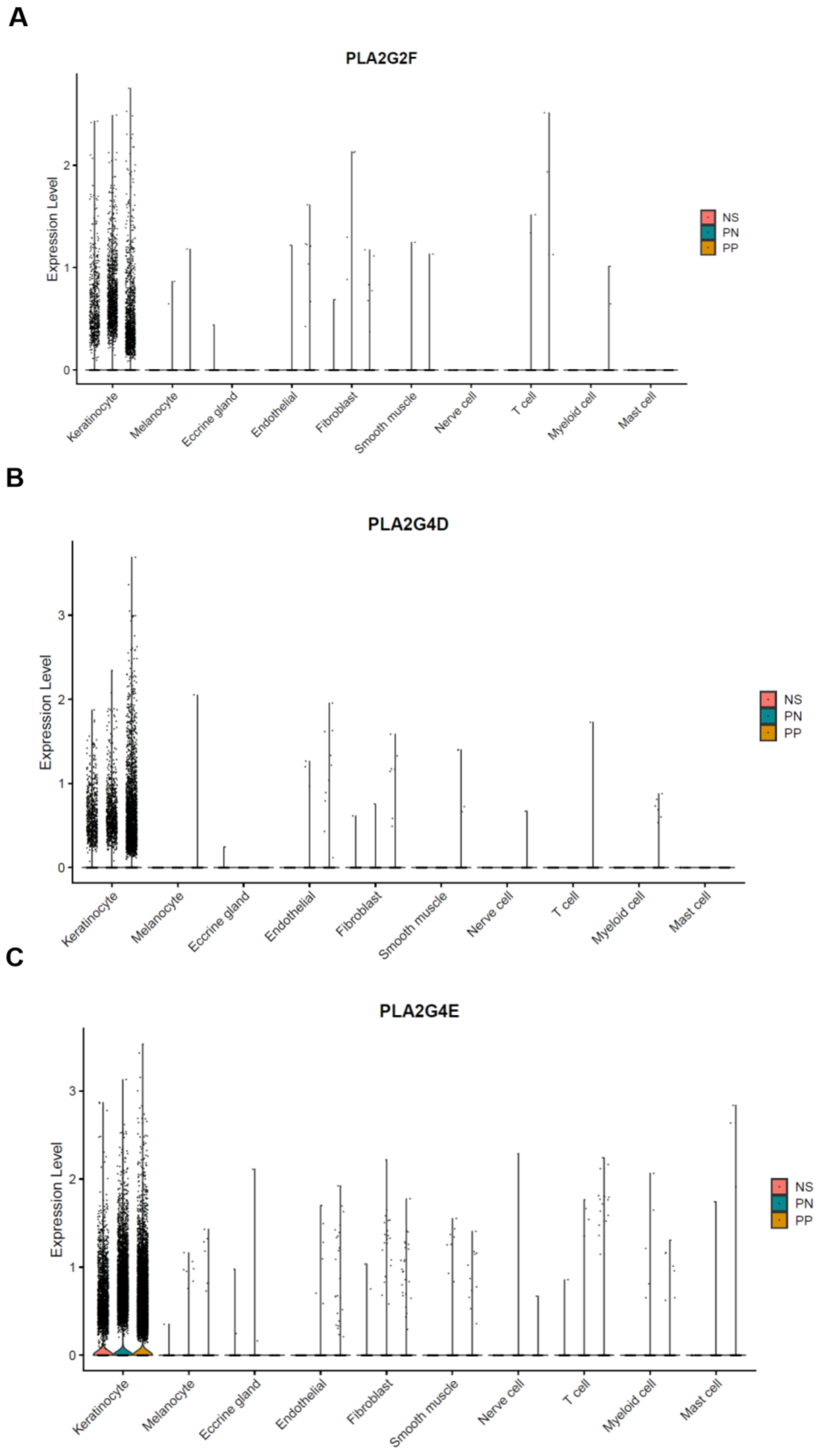

Figure S2. ScRNA-seq analysis of psoriatic lesional skin and controls for three PLA2 genes.

(A-C) ScRNA-seq analysis of psoriatic lesional skin (PP), non-lesional skin (PN), and normal controls (NS) demonstrates expression of the three PLA2s primarily within the epidermal keratinocytes.

Figure S3

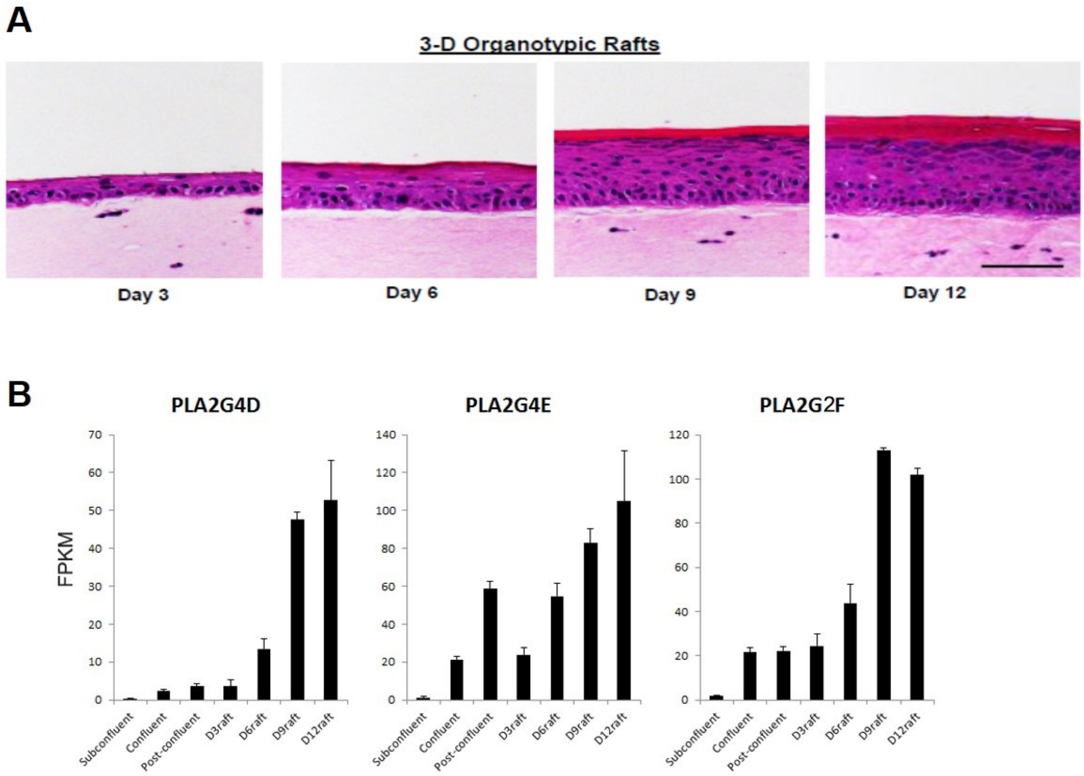

Figure S3. The expression level of PLA2G2F, PLA2G4D, and PLA2G4E in 3D human skin equivalents.

(A) The Hematoxylin and eosin staining of 3D human skin equivalents at indicated time. (B) The mRNA expression level of *PLA2G2F*, *PLA2G4D*, or *PLA2G4E* in the epidermis of 3D human skin equivalents.

Figure S4

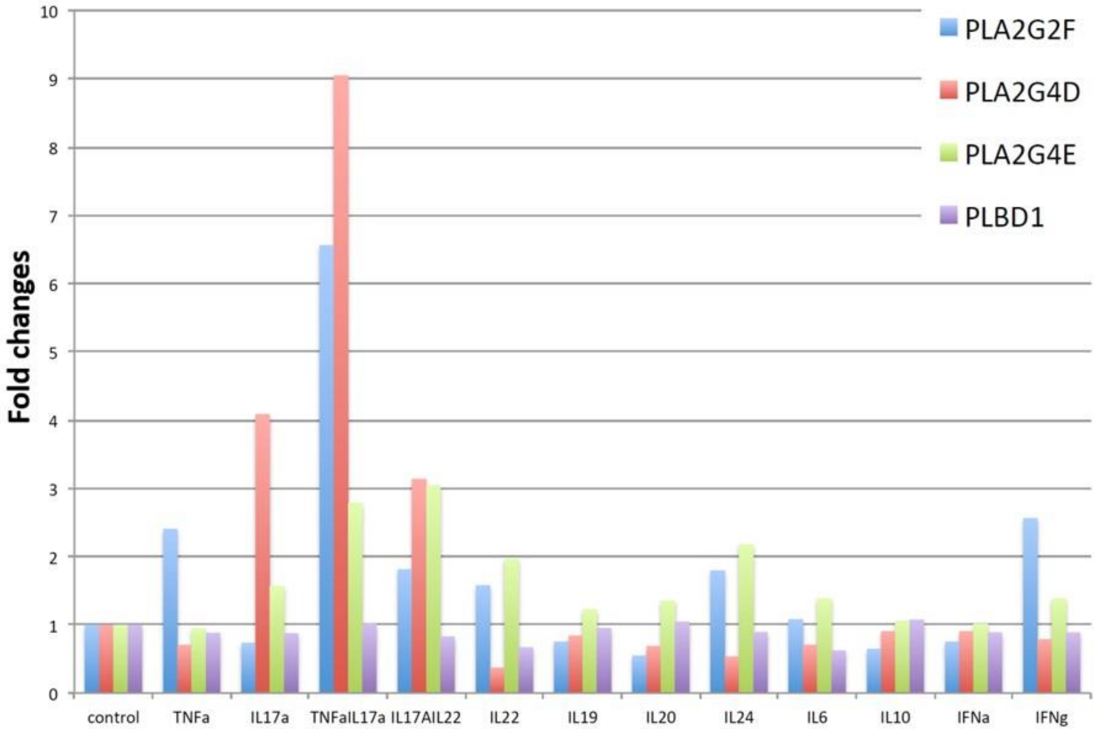

Figure S4. The expression level of PLA2G2F, PLA2G4D, and PLA2G4E in cytokine-treated keratinocytes.

QRT-PCR detected the mRNA expression level of *PLA2G2F*, *PLA2G4D*, or *PLA2G4E* in keratinocytes stimulated by a set of cytokines.

Figure S5

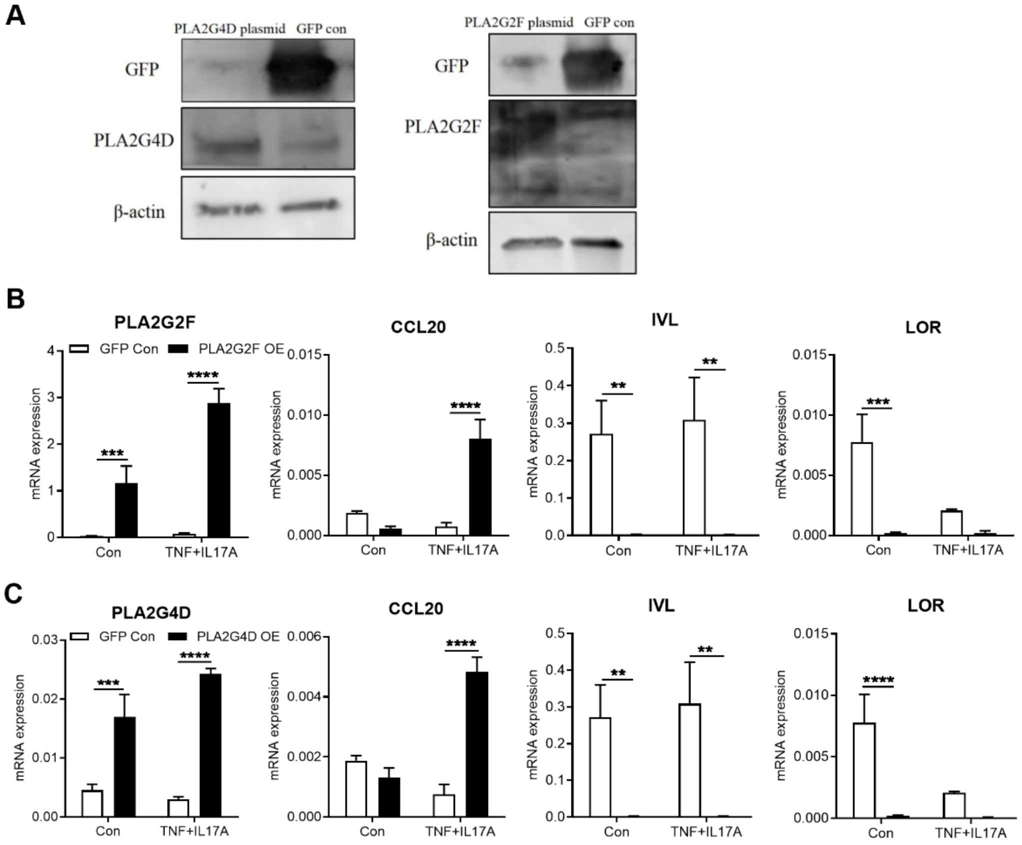

Figure S5. Overexpressing *PLA2G2F* or *PLA2G4D* modulates the immune response and skin barrier genes in keratinocytes.

(A) Western blat validates the efficiency of *PLA2G2F* or *PLA2G4D* overexpression in keratinocytes. (B, C) QRT-PCR showing the mRNA expression of immune response and skin barrier genes in *PLA2G2F* (B) or *PLA2G4D* (C) overexpressed keratinocytes. Two-way ANOVA. Data are presented as means ± SEM (n=3). *P < 0.05, **P < 0.01, ***P < 0.001, ****P < 0.0001.

Figure S6

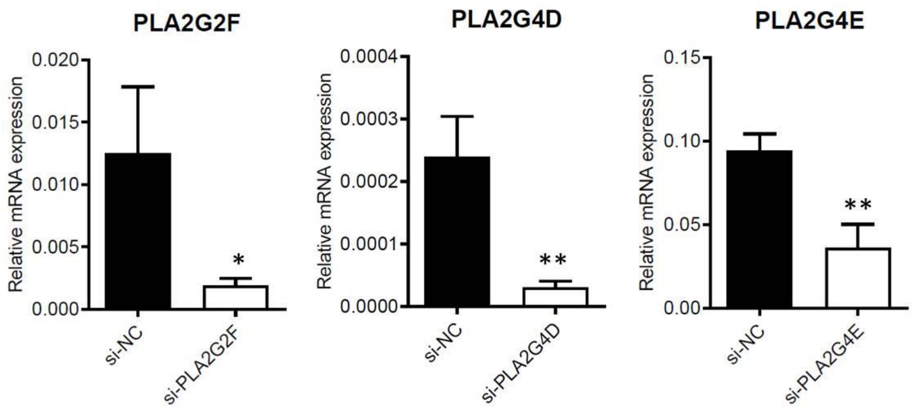

Figure S6. The knockdown efficiency of PLA2G2F, PLA2G4D, and PLA2G4E in keratinocytes. The siRNAs targeting *PLA2G2F, PLA2G4D, and PLA2G4E* were transfected in keratinocytes, and qRT-PCR validated the knockdown efficiency.

Figure S7

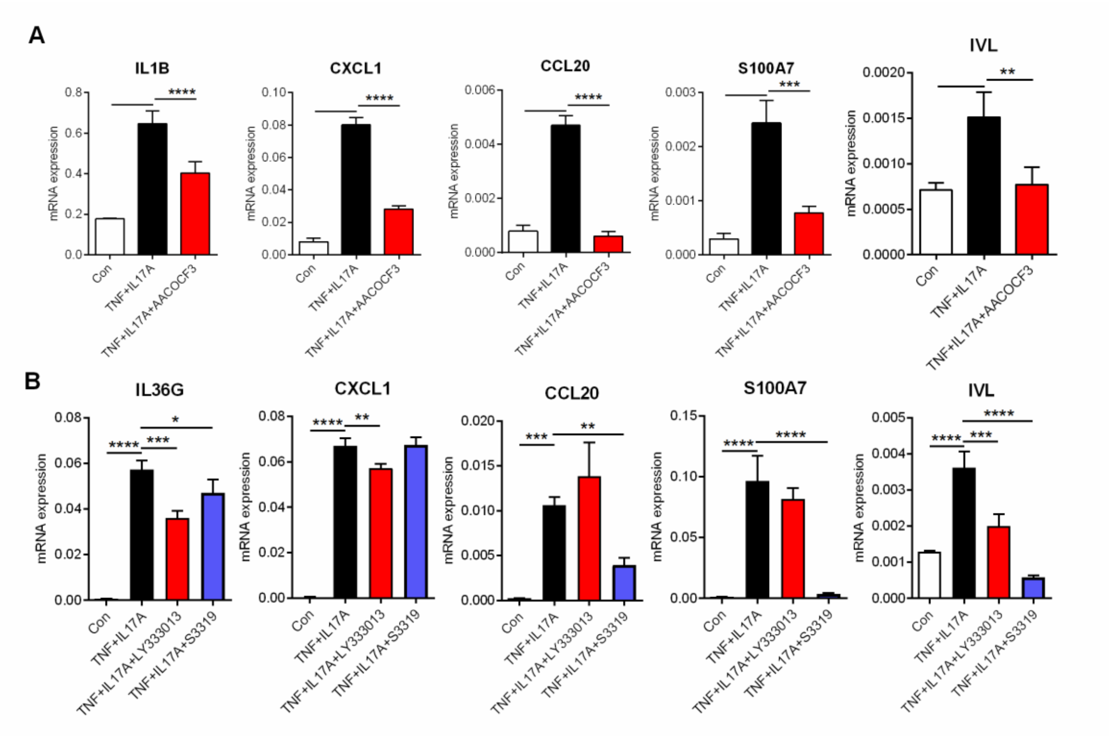

Figure S7. Pharmacological inhibiting sPLA2 or cPLA2 alleviates inflammatory responses and helps normalize differentiation in *vitro*.

(A, B) QRT-PCR showing the mRNA expression of immune response and skin barrier genes in keratinocytes that pre-treated with sPLA2 inhibitor (AACOCF3) (A) or cPLA2 inhibitors (LY333013 and S3319). One-way ANOVA. Data are presented as means ± SEM (n=3). *P < 0.05, **P < 0.01, ***P < 0.001, ****P < 0.0001.

Figure S8

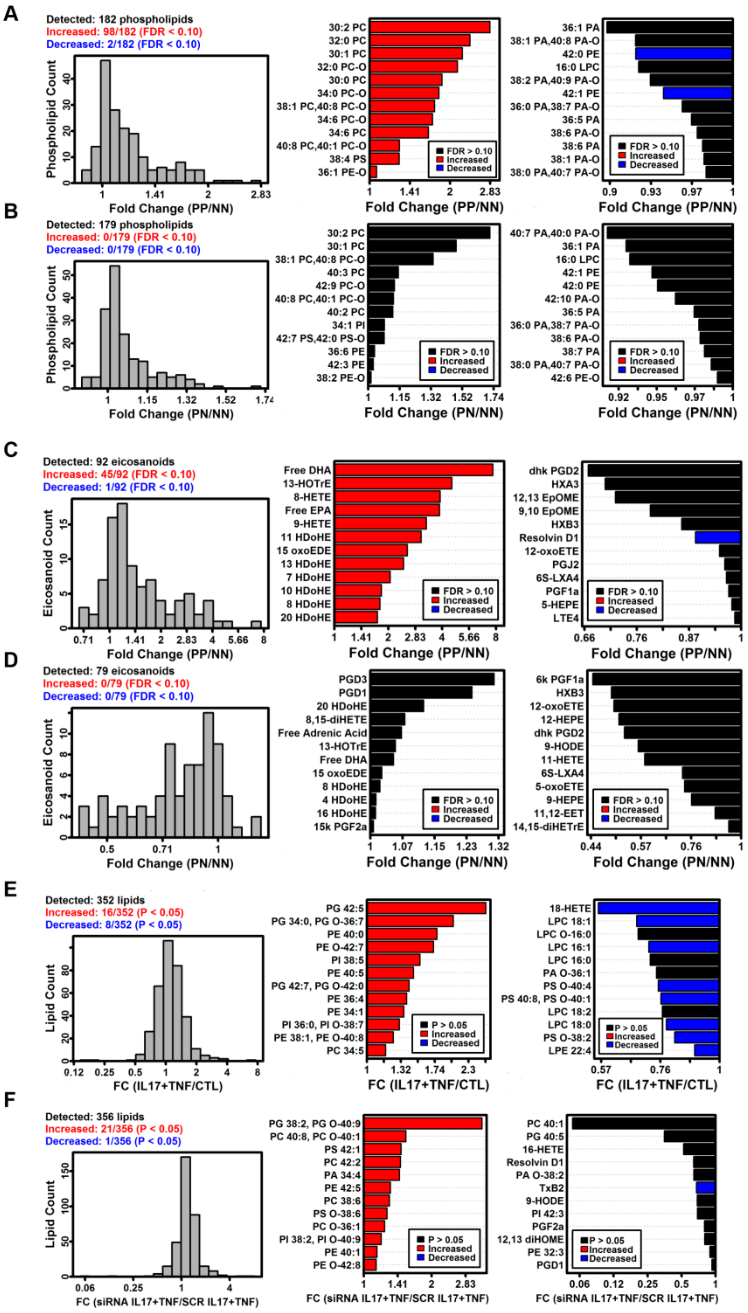

Figure S8. Lipids with most strongly altered abundance in comparisons among PP and NC skin samples, and in TNF+IL-17A stimulated keratinocytes.

(A) Distribution of fold-change estimates (PP/NN) (left panel). Phospholipids most strongly increased and decreased in PP vs. NN skin (right two panels). (B) Distribution of fold-change estimates (PN/NN) (left panel). Phospholipids most strongly increased and decreased in PN vs. NN skin (right two panels). (C) Distribution of fold-change estimates (PP/NN) (left panel). Eicosanoids most strongly increased and decreased in PP vs. NN skin (right two panels). (D) Distribution of fold-change estimates (PN/NN) (left panel). Eicosanoids most strongly increased and decreased in PN vs. NN skin (right two panels). Red and blue bars denote significantly increased and decreased lipids, respectively ($P < 0.05$). (E) Distribution of

fold-change estimates (TNF+IL17A/CTL) (left panel). Lipids most strongly increased and decreased in TNF+IL17A-treated KCs (right two panels). (F) Distribution of fold-change estimates (siRNA TNF+IL17A/SCR TNF+IL17A) (left panel). Lipids most strongly increased and decreased by siRNA knockdown (TNF+IL17A-treated cells) (right two panels). PP: psoriatic lesional skin; PN: psoriatic non-lesional skin; NN: normal controls.

Figure S9

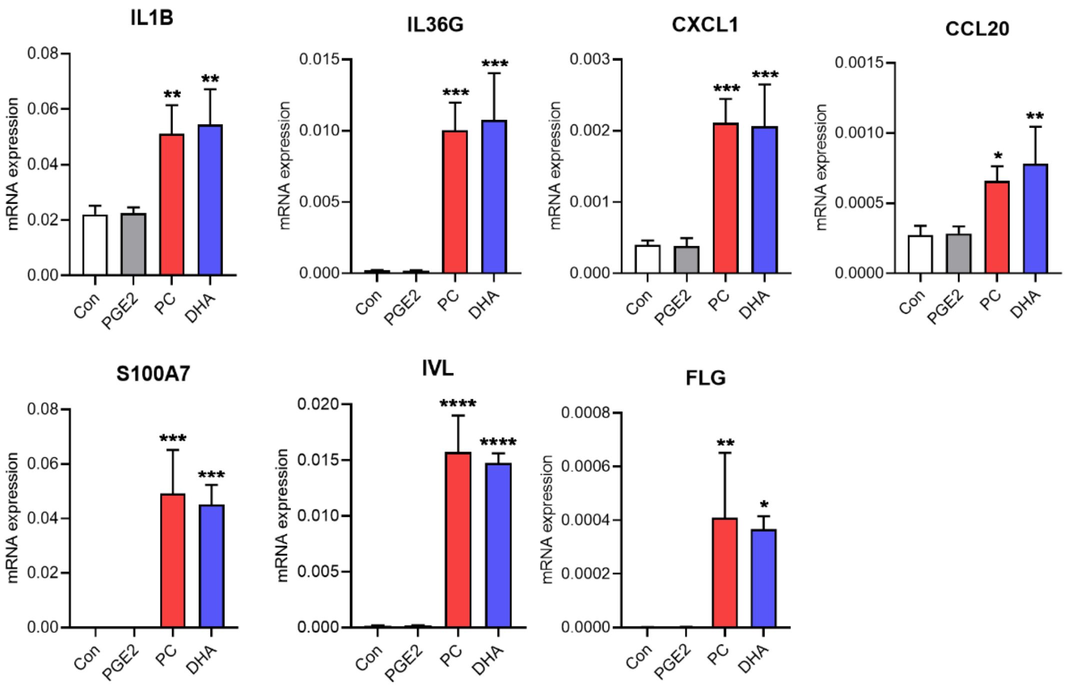

Figure S9. The proinflammatory role of some PLA2s-regulated lipids. QRT-PCR showing that the immune response and skin barrier genes in keratinocytes that stimulated by Phosphorylcholine (PC) and Docosahexaenoic Acid (DHA). One-way ANOVA. Data are presented as means ± SEM (n=3). $*P < 0.05$, $**P < 0.01$, $***P < 0.001$, $****P < 0.0001$.

Figure S10

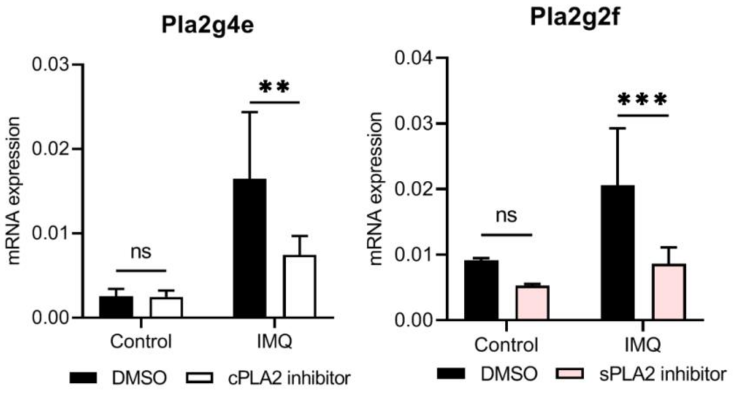

Figure S10. Inhibitors of PLA2s reduce the mRNA expression level of Pla2s in IMQ-induced mice.

QRT-PCR showing that the efficiency of topical PLA2 inhibitors in mouse epidermis. One-way ANOVA. Data are presented as means ± SEM (n=3). $**P < 0.01$, $***P < 0.001$, ns, no significance.
